# Frailty as a risk factor for postoperative complications in adult patients with degenerative scoliosis administered posterior single approach, long-segment corrective surgery: a retrospective cohort study

**DOI:** 10.1186/s12891-021-04186-9

**Published:** 2021-04-06

**Authors:** Bin Li, Xianglong Meng, Xinuo Zhang, Yong Hai

**Affiliations:** 1 Department of Orthopedic surgery, Beijing Chaoyang Hospital, Capital Medical University, No. 8 Gong Ti Nan Lu, Chaoyang District, Beijing, 100020 China; 2 Department of Orthopedics, Beijing Haidian Hospital, Haidian section of Peking University Third Hospital, Beijing, 100080 China

**Keywords:** Frailty, Adult degenerative scoliosis, Complications, Long-segment fusion

## Abstract

**Background:**

With the population aging worldwide, adult degenerative scoliosis (ADS) is receiving increased attention. Frailty, instead of chronological age, is used for assessing the patient’s overall physical condition. In ADS patients undergoing a posterior approach, long-segment corrective surgery, the association of frailty with the postsurgical outcomes remains undefined.

**Methods:**

ADS patients who underwent a posterior approach, long-segment fusion at the Department of Orthopedics, Beijing Chaoyang Hospital, Capital Medical University (CMU), Beijing, China, in 2014–2017 were divided into the frailty and non-frailty groups according to the modified frailty index. Major postoperative complications were recorded, including cardiac complications, pneumonia, acute renal dysfunction, delirium, stroke, neurological deficit, deep wound infection, gastrointestinal adverse events, and deep vein thrombosis. Radiographic measurements and health-related quality of life (HRQOL) parameters were recorded preoperatively and at 2 postoperative years.

**Results:**

A total of 161 patients were included: 47 (29.2%) and 114 (70.8%) in the frailty and non-frailty groups, respectively. Major postoperative complications were more frequent in the frailty group than the non-frailty group (29.8% vs. 10.5%, *P* = 0.002). Multivariable logistic regression analysis showed that frailty was independently associated with major complications (adjusted odds ratio [aOR] = 2.77, 95% confidence interval [CI] 1.12–6.89, *P =* 0.028). Radiographic and HRQOL parameters were improved at 2 years but with no significant between-group differences.

**Conclusions:**

Frailty is a risk factor for postoperative complications in ADS after posterior single approach, long-segment corrective surgery. Frailty screening should be applied preoperatively in all patients to optimize the surgical conditions in ADS.

**Supplementary Information:**

The online version contains supplementary material available at 10.1186/s12891-021-04186-9.

## Background

Adult scoliosis, reflected by a coronal Cobb angle of 10° or above in an adult individual, mainly includes adult idiopathic scoliosis (AdIS) and de novo adult degenerative scoliosis (ADS) and occurs during adulthood due to progressive degenerative changes [1]. ADS is detected in approximately 68% of asymptomatic individuals above age 60, and its prevalence increases with age [2]. With the population aging worldwide, ADS attracts increasing attention and since it imposes living and economic burdens on patients and their families. Conservative treatments, such as drug therapy, traction, massage, acupuncture, and epidural steroid injection, represent the first-line treatments for ADS, but surgical treatment is recommended in case of unsatisfactory conservative treatment [3]. Coronal Cobb angle > 30°, intervertebral lateral listhesis > 6 mm, apical rotation above grade II, and intercrest line below the lumbar 4/5 intervertebral space are factors predicting disease progression [4]. Aggravating scoliosis and neurological symptoms often result in severe chronic back pain, radiating leg pain, and neurogenic claudication in ADS patients [5].

Although long-segment corrective surgery alleviates pain and improves functional movement and quality of life, it is associated with a high rate of postoperative complications due to deformity heterogeneity and surgical complexity [6–8]. Therefore, future studies assessing ADS should perform pre-operative screening of populations at high risk of postoperative complications, determine related risk factors after surgical intervention, and develop effective perioperative management tools to reduce postoperative complications.

Frailty is a condition accompanying aging, characterized by declining physiological reserve capacity of nerves, muscles, metabolism, and immunity, leading to reduced ability to resist physical and psychological stress [9–11]. It is reflected by increased susceptibility and declined capability to maintain homeostasis. Previous studies [12–14] have suggested that frailty assessment has a certain value in screening high-risk patients before surgery. It has not been previously used in ADS patients treated by a posterior single approach, multi-segment corrective surgery. Therefore, the present study aimed to assess the association of frailty with postsurgical outcomes in ADS patients who underwent a posterior approach, long-segment corrective surgery.

## Methods

### Subjects

Consecutive patients with ADS treated surgically at the Department of Orthopedics, Beijing Chaoyang Hospital, Capital Medical University (CMU), Beijing, China, from January 1, 2014, to December 31, 2017, were included in this retrospective cohort study. The inclusion criteria were 1) ADS treated by a posterior single approach, long-segment corrective surgery, 2) age > 50 years, 3) coronal Cobb’s angle > 30° and fusion levels > 3 motion segments, 4) integrated preoperative and follow-up radiographic data, 5) complete preoperative and follow up functional evaluation data, and 6) follow-up > 2 years. The exclusion criteria were 1) previous operation of the thoracolumbar spine, 2) other scoliosis types, including ankylosing spondylitis, spinal tuberculosis, adolescent idiopathic scoliosis progressed in adulthood, and neuromuscular and congenital scoliosis, 3) spinal tumors, or 4) revision of previous ADS surgery. The study was approved by the Ethics Committee of Beijing Chaoyang Hospital (The Third Affiliated Hospital of Capital Medical University). Informed consent was waived due to the retrospective nature of the study.

### Frailty assessment and surgical data

Frailty was assessed by the modified frailty index (mFI) in all patients (Supplementary Table S1), based on data extracted from the electronic medical record system. The mFI included 11 items: history of diabetes mellitus, changes in daily activity, lung problems, history of congestive heart failure, history of myocardial infarction, history of percutaneous coronary intervention, cardiac surgery or angina, hypertension, peripheral vascular disease, clouding or delirium, transient ischemic attack, and cerebrovascular accident with deficit [15, 16]. The mFI is the proportion of the total number of items present in the patient’s preoperative history divided by 11 (i.e., the total number of items used in the assessment). Each item was given equal weight (scored 0 or 1) in the scoring of the index. Patients were identified as frail with an mFI score > 0.27. This cutoff point was based on previous reports [13, 17, 18]. After frailty assessment by the mFI scale, the patients were divided into the frailty and non-frailty groups. There were 47 (29.2%) in the frailty group and 114 (70.8%) in the non-frailty group.

Laboratory data, including albumin, total-cholesterol, creatinine, white blood cells, lymphocyte, hemoglobin and platelet, and surgical data, including operation duration, intraoperative blood loss, blood transfusion, fusion levels, decompression levels, and length of stay in hospital (LOS), were recorded. All the operations were performed by the same team and included posterior instrumentation, posterior column osteotomy, nerve decompression, and fusion.

### Radiographic measurements

Radiographic data included the Cobb angle of the curves and coronal vertical axis (CVA) in the coronal plane, pelvic incidence minus lumbar lordosis mismatch (PI-LL), and the sagittal vertical axis (SVA) in the sagittal plane. CVA was the distance between the C7 plumb line and the central sacral vertical line. Lumbar lordosis was reflected by the Cobb angle between the T12 upper endplate and the S1 endplate. Pelvic incidence was the angle between the perpendicular to the sacral plate and a line connecting the sacral plate’s center to the femoral head center. SVA was the distance between the C7 plumb and the sacrum’s posterior superior corner [19, 20]. All radiographic measurements were performed independently by two spinal surgeons to decrease intra-observer variability; the obtained values were averaged and used for analysis.

### Complication assessment and health-related quality of life

The primary outcome was the major postoperative complications. Patients with cardiac complications, pneumonia, delirium, stroke, neurological deficit, deep wound infection, acute renal dysfunction, gastrointestinal adverse events, and deep vein thrombosis (DVT) or pulmonary embolism (PE) were recorded, according to the classification method reported by Glassman [6]. Cardiac comorbidities included acute myocardial infarction, congestive heart failure, atrial fibrillation, and malignant arrhythmia. Gastrointestinal adverse events encompassed digestive tract hemorrhage and alimentary tract perforation. Acute renal failure refers to an increase in serum creatinine ≥26.5 μmol/L or > 1.5 times the baseline value within 48 h. The radiographic and HRQOL parameters were recorded preoperatively and at 2 years postoperatively.

### Statistical analyses

Continuous variables, including age, BMI, fusion levels, decompression levels, and Oswestry disability index (ODI), Japanese Orthopedic Association scores (JOA), visual analog scale (VAS) for back pain, and Scoliosis Research Society-22 questionnaire (SRS-22) scores, are presented as means ± standard deviations (SDs) and were tested for normality using the Kolmogorov-Smirnov test. They were analyzed using the Student’s *t*-test or the Mann-Whitney U-test according to normality. Categorical data, such as sex and smoking, are presented as proportions and were analyzed using the chi-square and Fisher’s exact tests. Logistic analysis was performed to investigate the risk factors for complications. Major postoperative complications were recorded and described as proportions. Univariable and multivariable logistic regression analyses were carried out to investigate the possible association between frailty and major complications. The odds ratios (ORs) and 95% confidence intervals (CIs) were estimated after adjustment for each covariable in the univariable analyses. SPSS 22.0 (IBM Corp., Armonk, NY, USA) was used for data analysis. Two-tailed *P* < 0.05 was considered statistically significant.

## Results

### Patients characteristics

A total of 161 ADS patients were included. Their mean age was 66.27 ± 8.45 (50–83) years. The minimum follow-up time was 24 months. The patients in the frailty group were older compared with the non-frailty group (68.79 ± 7.88 vs. 65.23 ± 8.48 years, *P* = 0.015). Average intraoperative blood loss was significantly higher in the frailty group than the non-frailty group (1021.28 ± 392.28 vs. 871.49 ± 340.61 ml, *P* = 0.016). The average hospital stay was significantly longer in the frailty group than the non-frailty group (15.60 ± 3.20 vs. 13.89 ± 2.71 days, *P* = 0.001). The patients in non-frailty group had higher level of albumin (38.32 ± 3.52 vs. 36.13 ± 2.54 g/L) and hemoglobin (119.96 ± 16.30 vs. 114.36 ± 15.11 g/L) than those of patients in frailty group (all *p* < 0.05). No statistically significant differences were observed in the medical histories and the level of total-cholesterol, creatinine, white blood cells, lymphocyte and platelet between the two groups (Table 1).

**Table 1:** Patient characteristics

	Frailty group (*n* = 47)	Non-frailty group (*n* = 114)	*p* value
Age (y)	68.79 ± 7.88	65.23 ± 8.48	0.015
Gender, male (%)	21 (44.7%)	49 (43.0%)	0.843
BMI (kg/m^2^)	24.43 ± 2.24	24.91 ± 2.24	0.210
**Medical history**			
Hypertension, n (%)	22 (46.8%)	34 (29.8%)	0.040
Diabetes, n (%)	12 (25.5%)	17 (14.9%)	0.111
Cerebrovascular disease, n (%)	3 (6.4%)	5 (4.4%)	0.693
Respiratory disease, n (%)	6 (12.8%)	16 (14.0%)	0.831
Heart disease, n (%)	8 (17.0%)	11 (9.6%)	0.191
Smoking, n (%)	15 (31.9%)	30 (26.3%)	0.472
**Laboratory test**			
Albumin (g/L)	36.13 ± 2.54	38.32 ± 3.52	< 0.01
Total-cholesterol (mmol/L)	4.41 ± 0.88	4.19 ± 0.99	0.191
Creatinine (μmol/L)	130.45 ± 37.32	120.74 ± 35.10	0.119
White blood cells (*10^9^/L)	6.48 ± 1.47	6.43 ± 1.52	0.841
Lymphocyte (%)	27.67 ± 7.58	27.48 ± 8.61	0.897
Hemoglobin (g/L)	114.36 ± 15.11	119.96 ± 16.30	0.045
Platelet (*10^9^/L)	206.89 ± 57.25	203.82 ± 53.99	0.748
**Surgery data**			
Fusion levels	6.57 ± 1.91	6.18 ± 1.85	0.220
Decompression levels	2.32 ± 1.04	2.07 ± 1.03	0.166
Intra-operative blood loss (ml)	1021.28 ± 392.28	871.49 ± 340.61	0.016
Blood transfusion, n (%)	14 (29.8%)	24 (21.1%)	0.235
Operative time (min)	270.43 ± 37.06	260.18 ± 38.89	0.125
Hospital stay (day)	15.60 ± 3.20	13.89 ± 2.71	0.001

### Major postoperative complications

Major postoperative complications were remarkably more frequent in the frailty group (14/47, 29.8%) compared with the non-frailty group (12/114, 10.5%) (*P* = 0.003, Table 2). Univariable analyses were first performed to identify potential factors associated with major complications. The associated factors included frailty, age, smoking, hypertension, and fusion levels, all with *P* < 0.10. The latter parameters were entered in the multivariable logistic regression analysis to determine the possible risk factors for major postoperative complications. After adjustment for covariables, including age, smoking, fusion levels, hypertension, frailty was an independent risk factor for major postoperative complications (adjusted OR = 2.77, 95%CI: 1.12–6.89, *P* = 0.028) (Table 3).

**Table 2:** Complications in the frailty and non-frailty groups

	Frailty group (n = 47)	Non-frailty group (n = 114)	*p* value
Major peri-operative complications, n (%)	14 (29.8%)	12 (10.5%)	0.003
Cardiac complications, n (%)	2 (4.3%)	1 (0.9%)	0.204
Pneumonia, n (%)	2 (4.3%)	2 (1.8%)	0.581
Acute renal failure, n (%)	3 (6.4%)	1 (0.9%)	0.075
Stroke, n (%)	1 (2.1%)	1 (0.9%)	0.500
Neurological deficit, n (%)	3 (6.4%)	2 (1.8%)	0.149
Deep wound infection, n (%)	2 (4.3%)	3 (2.6%)	0.630
Gastrointestinal adverse events, n (%)	2 (4.3%)	2 (2.6%)	0.581
Deep vein thrombosis, n (%)	1 (2.1%)	1 (1.8%)	0.500
Delirium, n (%)	3 (6.4%)	1 (1.8%)	0.075

**Table 3:** Univariate and multivariate logistic regression analyses of major post-operative complications

	Odds ratio (95% CI)	*p* value	Odds ratio (95% CI)	*p* value
	Univariate analysis		Multivariate analysis	
Frailty	3.61 (1.52–8.57)	0.004	2.77 (1.12–6.89)	0.028
Gender	1.56 (0.65–3.74)	0.322		
Age	1.05 (1.00–1.11)	0.067	1.05 (0.99–1.11)	0.096
BMI	1.02 (0.84–1.23)	0.875		
Smoking	2.17 (0.91–5.20)	0.079	2.25 (0.88–5.75)	0.090
Lung disease	0.80 (0.22–2.91)	0.731		
Hypertension	2.14 (0.91–5.00)	0.079	2.02 (0.81–5.08)	0.133
Diabetes	1.90 (0.71–5.04)	0.202		
Cardiovascular disease	1.45 (0.44–4.80)	0.538		
Fusion level	1.28 (1.03–1.59)	0.029	1.28 (1.01–1.63)	0.042
Albumin	0.97 (0.85–1.10)	0.620		
Total-cholesterol	1.07 (0.69–1.64)	0.772		
Creatinine	1.01 (0.99–1.02)	0.176		
White blood cells	1.07 (0.81–1.41)	0.647		
Lymphocyte	0.97 (0.92–1.02)	0.223		
Hemoglobin	0.98 (0.96–1.01)	0.236		
Platelet	1.00 (0.99–1.01)	0.286		

### Radiological parameters and HRQOL

Radiographic parameters were similar in the frailty and non-frailty groups, including Cobb, pelvic incidence minus lumbar lordosis (PI-LL), the coronal vertical axis (CVA), and the sagittal vertical axis (SVA), preoperatively and at 2 years postoperatively (all *P* > 0.05, Table 4).

**Table 4:** Radiographic parameters in the frailty and non-frailty groups

	Frailty group (n = 47)	Non-frailty group (n = 114)	*p* value
Pre-operative Cobb (°)	37.28 ± 4.21	36.11 ± 4.56	0.135
Cobb at follow-up (°)	10.49 ± 1.35	10.05 ± 1.92	0.157
Pre-operative CVA (mm)	40.45 ± 9.95	41.29 ± 10.42	0.637
CVA at follow-up (mm)	10.74 ± 5.64	10.12 ± 5.79	0.533
Pre-operative PI-LL (°)	30.30 ± 4.00	29.77 ± 5.04	0.525
PI-LL at follow-up (°)	10.06 ± 2.69	9.91 ± 3.07	0.768
Pre-operative SVA (mm)	72.13 ± 43.89	75.61 ± 30.45	0.565
SVA at follow-up (mm)	23 ± 16.63	20.41 ± 15.23	0.342

*PI-LL* pelvic incidence minus lumbar lordosis, *CVA* coronal vertical axis, *SVA* sagittal vertical axis

Similarly, the patient’s HRQOL parameters showed no significant differences between the frailty and non-frailty groups, including ODI, JOA, VAS for back pain, and SRS-22 scores (Table 5).

**Table 5:** HRQOL in the frailty and non-frailty groups

	Frailty group (n = 47)	Non-frailty group (n = 114)	*p* value
Pre-operative ODI	62.83 ± 3.92	62.01 ± 3.93	0.230
ODI at follow-up	26.40 ± 4.12	25.54 ± 3.97	0.218
Pre-operative VAS	6.53 ± 1.32	6.17 ± 1.47	0.142
VAS at follow-up	2.38 ± 0.97	2.18 ± 0.98	0.222
Pre-operative JOA	9.72 ± 2.08	9.73 ± 1.92	0.990
JOA at follow-up	20.43 ± 1.85	20.30 ± 2.03	0.711
Pre-operative SRS-22	41.79 ± 6.34	43.51 ± 5.91	0.102
SRS-22 at follow-up	86.34 ± 9.09	87.87 ± 8.29	0.303

*ODI* Oswestry Disability Index, *JOA* Japanese Orthopaedic Association scores, *VAS*: Visual Analogue Scale

## Discussion

Surgery for ADS has undeniable advantages in eliminating or relieving the disease’s symptoms, especially in patients in whom conservative treatment fails. The present study suggests that frailty is a risk factor for postoperative complications in ADS after posterior single approach, long-segment corrective surgery. ADS surgery is technically complex and diverse, focusing on the restoration of spinal alignment and the decompression of neural elements, with a high risk of complications [21, 22]. In this study, the major complication rate was 16.1% (26/161), supported by previous reports [21, 22]. Such a relatively high rate of complications associated with surgery for adult spinal deformities calls for identifying the associated risk factors.

The rate of complications in the non-frailty group was high. Patients with ADS represent a heterogeneous group of patients in terms of affected spinal segments and deformity angles. In addition, since it is an age-related disease, the patients with ADS are also heterogeneous in comorbidities and chronic conditions. Since frailty is defined as an mFI score > 0.27 (or > 3 positive items out of 11) [13, 17, 18], it still leaves the patients in the non-frailty group with 0 to 2 comorbidities among history of diabetes mellitus, changes in daily activity, lung problems, history of congestive heart failure, history of myocardial infarction, history of percutaneous coronary intervention, cardiac surgery or angina, hypertension, peripheral vascular disease, clouding or delirium, transient ischemic attack, and cerebrovascular accident with deficit [15, 16]. Since any one of these conditions can increase the surgical risk, this might explain why the rate of complications in the non-frailty group is still high. The reported incidence of complications after corrective spinal surgeries ranges from 33 to 70% [6, 23, 24].

Two major frailty models include the frailty phenotype and the deficit accumulation model, which is also termed the frailty index. Originally, the deficit accumulation model included 92 variables [10], making its application difficult. In 2013, Velanovich et al. [16] proposed the modified frailty index (mFI) that consists of only 11 variables that can be easily extracted by simple electronic medical record review and physical examination.

Robinson et al. [25] reported increased mFI as a predictor of increased mortality at 1 year. Traven and collaborators [26, 27] reported that frailty is a robust predictor of multiple complications and mortality after primary hip and knee replacement and total shoulder arthroplasty. Others obtained similar findings in anterior lumbar interbody fusion surgery [28] and lumbar fusion [29]. Leven et al. [30] reported frailty as a strong predictor of complications, mortality, and reoperation in adult spinal deformity patients treated surgically, although the type of spinal deformity and the surgical procedures were not disclosed. The present study, to our knowledge, is the first investigation of the associations of frailty with the outcomes of posterior single approach long-segment corrective surgery in adult degenerative scoliosis, which could minimize the bias caused by different disorder types and surgical methods. In the present study, frailty (mFI > 0.27) was an independent risk factor for major postoperative complications. Frailty, instead of individual medical comorbidities or other single risk factors, strongly predicted postoperative complications. Indeed, frailty is a comprehensive concept containing major comorbidities, therefore representing the dominant predictive factor of postoperative complications. These findings suggest that frailty assessment should be applied during the risk stratification process.

As shown above, radiographic measurements and HRQOL after surgery in the frailty and non-frailty groups were significantly improved, with no statistically significant differences between the two groups, despite the elevated rate of complications in the frailty group. Similar data have been reported by others [31, 32]. The current patients were all administered posterior approach corrective surgery, which might partially explain the satisfactory outcome. Therefore, a single posterior approach corrective surgery might be safe and effective in ADS patients with frailty.

Before the study, we hypothesized that frailty would increase postoperative complications and that these complications would further affect the postoperative outcomes (including radiological parameters and HRQOL). Hence, the mFI was used for risk assessment to determine whether the patients could undergo surgery safely. The study’s final results strongly suggest that frailty did lead to a significant increase in postoperative complications. Surprisingly, there were no significant differences in postoperative clinical efficacy indexes between the frailty and non-frailty groups. These results are supported by previous studies [31, 32]. The exact reason cannot be determined by the present study, but it could be hypothesized that once the critical period of complication management has gone by and the complications are managed, the clinical efficacy of the surgery at 2 years is the same. Still, the present study could not examine the long-term outcomes because of data availability in two years of follow-up, and future studies should examine the short- and long-term outcomes after surgery in frail patients.

Based on the study results, we believe that it is not appropriate to use the mFI to determine whether patients with ASD can receive surgery. Still, patients with frailty do have a higher risk of postoperative complications, which will inevitably cause additional burden to the patients, whether physically, psychologically, economically, or in the subsequent treatment experience. Therefore, we believe that preoperative evaluation, management, and frailty improvement might reduce the risk of postoperative complications. Effective preoperative communication with the patients or their families could be conducted according to the preoperative evaluation results, which is conducive to establishing a good doctor-patient relationship and improving the outcomes. The study had several potentially important limitations. First, it was a retrospective trial, and future prospective multicenter studies should comprehensively assess complications in such patients. Second, the sample size was relatively small. The individual complications were not evaluated, and the relationship between frailty increase and complications could be determined. Further larger studies are therefore warranted.

## Conclusion

Frailty is associated with major postoperative complications in ADS patients who underwent posterior approach long-segment corrective surgery. A notable improvement in spine alignment and HRQOL was achieved in patients with frailty, with no statistically significant differences between the frail and non-frailty groups. These findings suggest that ADS with frailty should not be surgically contraindicated; instead, frailty screening should be applied preoperatively and universally to optimize treatment in adult degenerative scoliosis.

## Supplementary Information

**Additional file 1.**

## Data Availability

The datasets used and/or analyzed during the current study are available from the corresponding author on reasonable request.

## Abbreviations

AdIS: Adult idiopathic scoliosis
ADS: Adult degenerative scoliosis,
CVA: Coronal vertical axis
DVT: Deep vein thrombosis
HRQOL: Health-related quality of life
LOS: Length of stay in hospital
mFI: Modified frailty index
PI-LL: Pelvic incidence minus lumbar lordosis mismatch
PE: Pulmonary embolism
SVA: Sagittal vertical axis
