# Supplementary material for: Frailty as a risk factor for postoperative complications in adult patients with degenerative scoliosis administered posterior single approach, long-segment corrective surgery: a retrospective cohort study
Source: BMC Musculoskelet Disord. 2021 Apr 6;22:333. doi: 10.1186/s12891-021-04186-9 (PMC8025316; doi:10.1186/s12891-021-04186-9)
Supplement: Supplementary file 1 — Additional file 1. [file 12891_2021_4186_MOESM1_ESM.docx]

**Supplementary Table S1. The modified frailty index (mFI)**.

| History of diabetes mellitus |
| --- |
| Changes in everyday activity |
| Lung problem (including COPD and current pneumonia) |
| History of congestive heart failure |
| History of myocardial infarction (within 6 months before the surgery) |
| History of percutaneous coronary intervention, cardiac surgery, or angina (1 month before the surgery) |
| Hypertension requiring medicine |
| Peripheral vascular disease or rest pain |
| Clouding or delirium |
| TIA or cerebrovascular accident without current residual deficit |
| Cerebrovascular accident with deficit |
| COPD=chronic obstructive pulmonary disease. TIA=transient ischemic attack |
